# Comparative effectiveness of robotic and video assisted thoracic surgery for lung cancer across key outcomes

**DOI:** 10.1016/j.isci.2026.114898

**Published:** 2026-02-03

**Authors:** Guohang Shen, Ruoyan Wang, Jingyi Xu, Jingwen Chen, Ruoyu Chen, Yiru Wang, Xianquan Zhang, Kaiyong Wang, Yupei Dai, Junfeng Li

**Affiliations:** 1 Department of Thoracic Surgery, Beijing Anzhen Nanchong Hospital of Capital Medical University & Nanchong Central Hospital, Nanchong, Sichuan 637000, China; 2 North Sichuan Medical College, No.234 Fujiang Road, Shunqing District, Nanchong, Sichuan 637000, China; 3 School of Basic Medicine and Forensic Medicine, North Sichuan Medical College, No.234 Fujiang Road, Shunqing District, Nanchong, Sichuan 637000, China; 4 Department of Clinical Medicine, Ningxia Medical University, Yinchuan, Ningxia Hui Autonomous Region 750004, China

**Keywords:** Health sciences, Medicine, Surgery, Oncology

## Abstract

Robotic-assisted thoracic surgery (RATS) and video-assisted thoracoscopic surgery (VATS) are widely used for minimally invasive resection of pulmonary tumors, but they differ in technical capabilities and resource demands. We conducted a PRISMA-based systematic review and meta-analysis of randomized and cohort studies identified by searching PubMed, Embase, Web of Science, Scopus, and the Cochrane Library through June 2025. Across 25 studies including 41,417 patients, RATS was associated with lower conversion to thoracotomy rates and less blood loss, more extensive lymphadenectomy, higher R0 resection rates, and shorter chest tube duration, ICU stay, and hospital stay. Pneumonia and atrial fibrillation were less frequent, whereas overall complications, pneumothorax, and 30- and 90-day mortality were similar. Hospitalization costs were higher, and pooled survival data suggested a possible long-term advantage. These findings clarify platform tradeoffs and motivate large randomized trials and cost-effectiveness analyses.

## Highlights

• RATS was compared with VATS for surgical treatment of non-small cell lung cancer
• R0 resection rates and lymph node yield were comparable between RATS and VATS
• Perioperative outcomes and major postoperative complications were similar
• Costs and 5-year overall survival were assessed to benchmark RATS versus VATS

## Graphical abstract

**Figure:**
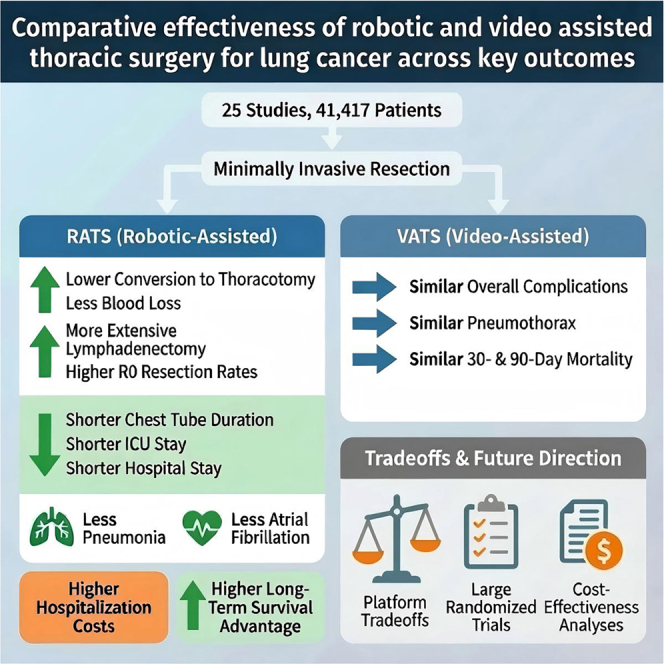

## Introduction

Cancer of the lung remains a leading cause of death worldwide and poses a considerable burden on global healthcare systems. Based on the Global Cancer Statistics 2022, lung cancer accounted for roughly 2.48 million incident cases worldwide in that year, equivalent to 12.4% of the total cancer burden and the highest among all tumor types. In the same year, lung cancer caused 1.82 million deaths, representing 18.7% of all cancer-related deaths and making it the foremost contributor to global cancer mortality.^1^ In clinical practice, surgical resection remains the primary treatment for resectable early-stage non-small cell pulmonary carcinoma (NSCLC), continuing to serve as the cornerstone of curative care. Among surgical approaches, lobectomy with systematic lymph node dissection is widely regarded as the standard treatment, as it enables complete tumor removal and accurate pathological staging, thereby guiding postoperative management and improving prognosis.^2^

Since its introduction in the mid-1990s, video-assisted thoracoscopic surgery (VATS) for lobectomy has been shown in numerous studies to provide perioperative benefits, including fewer complications, shorter hospital stays, better postoperative pain control, and faster recovery, while maintaining oncologic safety comparable to open thoracotomy. With ongoing advances in instruments and techniques, VATS has evolved from multiportal to uniportal approaches, aiming to further minimize surgical trauma and accelerate recovery. Nonetheless, limitations such as two-dimensional visualization, restricted instrument maneuverability, and a relatively steep learning curve remain significant challenges, particularly in complex anatomical regions or difficult lymph node dissections.^3^

Robotic-assisted thoracic surgery (RATS) was developed to overcome these limitations. By providing three-dimensional high-definition vision and articulating robotic arms, RATS enhances surgical precision and dexterity, offering particular advantages in complex dissections and potentially improving lymphadenectomy quality.^4^^,^^5^^,^^6^ Over the past decade, retrospective studies and systematic reviews have shown that perioperative outcomes—including complications, blood loss, length of stay, and short-term mortality—are largely comparable between RATS and VATS. Despite the initial longer operative times and higher associated costs, RATS may provide advantages in the thoroughness of lymph node dissection, the number of lymph node stations retrieved, and the potential for improved pathological staging.^5^^,^^7^ In addition, several studies suggest that the learning curve for RATS may be smoother than that for VATS, with efficiency and outcomes improving as institutional and surgeon experience increase.^8^^,^^9^

Despite these technical merits, the widespread adoption of RATS has been constrained by substantial equipment costs and the need for specialized training. By contrast, VATS—with lower resource requirements—has achieved broader global dissemination. Comparative studies of RATS versus VATS for lung cancer have yielded inconsistent findings. For example, Wu et al. reported that robotic anatomical lung resection provided superior 30-day mortality and disease-free survival compared with VATS;^6^ according to Ma et al., there were no meaningful differences between the two approaches in terms of postoperative complications, hospitalization duration, intraoperative blood loss, or 30-day mortality.^10^

Importantly, substantial heterogeneity persists across studies evaluating RATS and VATS in pulmonary resection, driven by differences in patient selection, tumor stage, surgical type (lobectomy versus sublobar resection), perioperative management, institutional experience, and learning curve. Moreover, ongoing innovations such as uniportal VATS and next-generation robotic systems further challenge the generalizability of earlier findings.^5^ Therefore, this study was designed to conduct an updated meta-analysis, incorporating the latest high-quality evidence to address these gaps. Unlike prior reviews,^6^^,^^11^^,^^12^ our analysis broadens the scope by systematically evaluating oncologic endpoints (R0 and R1 resection rates), short-term mortality (30- and 90-day), long-term survival (5-year overall survival), and hospitalization costs, in addition to perioperative outcomes. By integrating these parameters, this study aims to provide a comprehensive and contemporary evidence base to guide surgical decision-making and resource allocation in lung cancer management.

## Methods

### Literature search strategy

This systematic review and meta-analysis was conducted in accordance with the PRISMA guidelines and prospectively registered on PROSPERO (registration ID, CRD420251118017).^13^A comprehensive search was performed across PubMed, Scopus, Cochrane, Embase, and Web of Science to identify relevant publications up to June 2025. The search strategy combined keywords and medical subject headings, including “thoracoscopic,” “robot-assisted,” and “non-small cell lung cancer (NSCLC).” Only English-language publications were included. References from the identified clinical studies as well as meta-analyses were reviewed by hand to identify further relevant reports.

Two reviewers independently screened all records for eligibility. Any discrepancies between the reviewers were resolved through discussion, and unresolved disagreements were adjudicated by a third senior reviewer to ensure consistency in study selection. To avoid duplicate inclusion from overlapping cohorts, the most complete or most recent report was retained. Boolean operators (AND/OR) were applied to broaden the search, and multiple combinations of keywords were used repeatedly.

### Inclusion criteria and data extraction

Inclusion criteria were defined according to the PICOS framework (population, intervention, comparison, outcomes, and study design): population (P), patients undergoing surgical resection for pulmonary nodules or tumors; intervention (I), robotic-assisted thoracic surgery (RATS); comparison (C), video-assisted thoracoscopic surgery (VATS); outcomes (O), perioperative outcomes, postoperative complications, and cost-related endpoints; and study design (S), randomized controlled trials (RCTs) and cohort studies.^14^ Among the included studies, lobectomy accounted for over 90% of all procedures, whereas segmentectomy represented approximately 8.9% and wedge resections were rare. Therefore, the pooled estimates primarily reflect outcomes in lobectomy-dominant cohorts. Data extracted included demographic variables (age, sex, and body mass index), pulmonary function (FEV_1_ and DLCO), comorbidities (COPD and diabetes), tumor characteristics (size and histology), and surgical approaches (lobectomy and segmentectomy). Exclusion criteria were duplicate studies, non-comparative designs (e.g., case reports, reviews, conference abstracts, meta-analyses, or single-arm studies), non-English language, insufficient data, or low-quality methodology. In total, 19 outcome indicators were analyzed. These were pre-specified as follows: primary outcomes included conversion to thoracotomy, R0 resection rate, number of dissected lymph nodes, number of sampled lymph node stations, major postoperative complications (composite endpoint), specific complications (pneumonia, atrial fibrillation, and atelectasis), and 5-year overall survival; as secondary outcomes, we assessed intraoperative blood loss, time to chest tube removal, ICU admission days, hospital stay length, postoperative drainage, frequency of R1 resections, mortality at 30 and 90 days, operative duration, and aggregate hospitalization expenditures. To improve methodological transparency, we also reviewed the definitions and measurement methods of key continuous outcomes across the included studies and summarized the common reporting patterns. In most studies, drainage volume was reported as the cumulative fluid output prior to chest tube removal; chest tube duration was defined as the interval from intraoperative tube placement to postoperative removal; hospital stay was reported as the total number of days from admission for surgery until discharge criteria were met; and costs were reported in thousand US dollars for ease of comparison across studies. Exchange rates were applied where necessary. Although minor variations existed among individual studies, these patterns represent the predominant approaches and help clarify potential sources of variability.

### Quality assessment, publication bias, and statistical analysis

The Newcastle-Ottawa scale was used to evaluate methodological quality, with scores ≥7 considered acceptable and 8–9 classified as high quality. For non-randomized studies, risk of bias was additionally assessed using the ROBINS-I tool, covering seven domains (confounding, participant selection, classification of interventions, deviations from intended interventions, missing data, outcome measurement, and reporting). Each domain was judged as low, moderate, serious, or critical, with the overall rating determined by the highest level of risk; studies rated as serious or critical were regarded as low quality and included in sensitivity analyses. For studies reporting medians and interquartile ranges, means and standard deviations were estimated using the methods of Luo, McGrath, and Shi.^15^^,^^16^^,^^17^ A minority of continuous outcomes required median-to-mean conversion, and sensitivity analyses indicated that these transformations had minimal impact on heterogeneity patterns or effect estimates. Pooled effect sizes were expressed as ORs for binary outcomes and MDs for continuous outcomes, both with 95% confidence intervals CIs. Heterogeneity was assessed using the I^2^ statistic (<25% low, 25%–50% moderate, and >50% high). Random-effects models were used when I^2^ exceeded 50%, otherwise, fixed-effects models were applied. If extreme heterogeneity was observed (I^2^ > 75%), prediction intervals were additionally calculated, and sensitivity analyses were performed by excluding influential studies or those with a higher risk of bias. These decision rules for model selection and sensitivity analyses were pre-specified in the PROSPERO protocol, and no deviations from the registered analytic plan occurred. Review manager software (RevMan, version 5.4; Cochrane Collaboration, Oxford, UK) was employed for statistical analysis, and results were considered significant when two-sided *p* values were below 0.05, to ensure the accuracy and appropriateness of the statistical methods employed, the analysis was reviewed by a professional biostatistician.^18^^,^^19^ The study-level extracted values and the computation files (RevMan project file) have been provided in the supplementary materials to facilitate reproducibility.

## Results

### Study selection and characteristics

**Figure 1:**
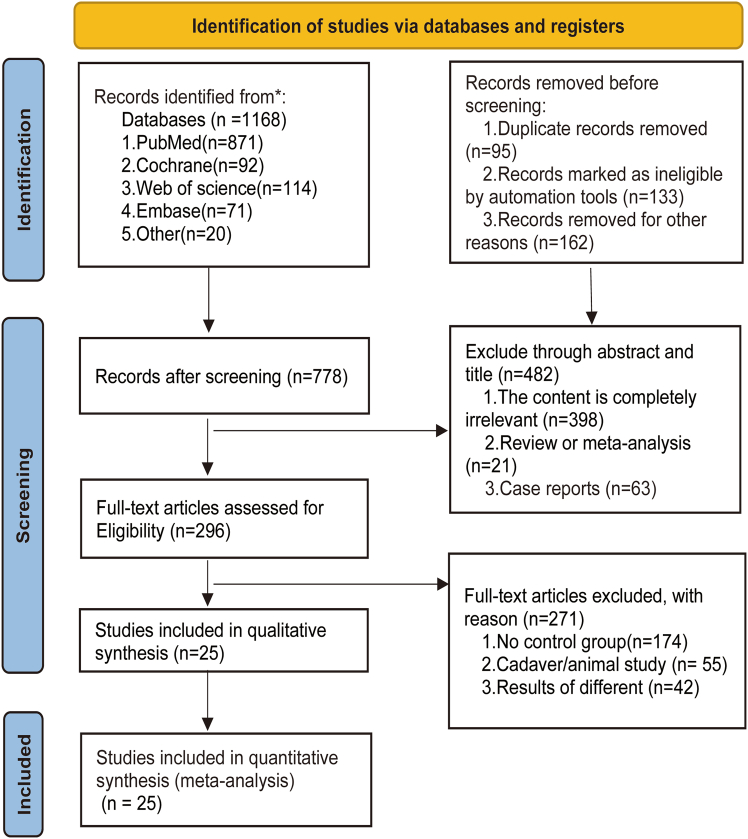
Research options flow chart

A total of 1,128 articles were identified through database and manual searches. After full-text screening (Figure 1 shows the flow diagram of study selection), 25 articles reporting 19 outcome indicators were included in the final analysis, encompassing 41,417 patients. Among these 12,645 patients in the experimental group underwent RATS, and 28,772 patients in the control group underwent VATS.^5^^,^^20^^,^^21^^,^^22^^,^^23^^,^^24^^,^^25^^,^^26^^,^^27^^,^^28^^,^^29^^,^^30^^,^^31^^,^^32^^,^^33^^,^^34^^,^^35^^,^^36^^,^^37^^,^^38^^,^^39^^,^^40^^,^^41^^,^^42^^,^^43^ Table 1 summarizes the baseline characteristics of the included studies, showing no significant differences between groups in variables such as sex, age, BMI, surgical method, pathological type, tumor size, and preoperative pulmonary function. Table 2 provides an overview of the 19 outcome measures, covering perioperative outcomes, postoperative survival, and hospitalization costs.

**Table 1:** Main characteristics of the included studies

Study ID (Year)	Group	Cases (*n*)	Age years (mean ± SD)	Female *n* (%)	BMI kg/m^2^ (Mean ± SD)	FEV1/L (Mean ± SD)	DLCO min/Kpa/*L* (mean ± SD)	COPD *n* (%)	DM *n* (%)	Tumor size mm (mean ± SD)	Lobectomy *n* (%)	Segmentectomy *n* (%)	Adenocarcinoma *n* (%)	Squamous cell carcinoma *n* (%)	Others *n* (%)
Giulia 2021	RATS	38	69 ± 8.3	17 (44.74)	27 ± 4.0	86 ± 25.0	76 ± 20.5	NA	NA	21.07 ± 10.02	36 (94.74)	2 (5.26)	26 (68.42)	6 (15.79)	6 (15.79)
	VATS	39	69 ± 7.3	16 (41.03)	26 ± 4.1	91 ± 24.8	76 ± 19.6			21.71 ± 12.31	37 (94.87)	2 (5.13)	31 (79.49)	3 (7.69)	5 (12.82)
Qianjun 2020	RATS	50	54.7 ± 10.3	35 (70.00)	23.7 ± 3.6	93.1 ± 16.5	95.3 ± 15.9	3 (6.00)	2 (4.00)	11.4 ± 3.5	0 (0)	50 (100)	16 (32.00)	NA	34 (68.00)
	VATS	80	57.7 ± 9.7	54 (67.50)	23.7 ± 2.8	89.4 ± 17.9	93.6 ± 22.1	6 (7.50)	4 (5.00)	12.4 ± 3.9	0 (0)	80 (100)	34 (42.50)		46 (57.50)
Rajwanth 2020	RATS	338	73 ± 8.0	190 (56.21)	NA	NA	NA	NA	NA	NA	312 (92.31)	26 (7.69)	215 (63.61)	93 (27.51)	NA
	VATS	1230	72 ± 7.0	688 (55.93)							1,127 (91.63)	103 (8.37)	812 (66.02)	295 (23.98)	123 (10.00)
Seder 2022	RATS	2133	66.62 ± 9.1	1,222 (57.29)	34.9 ± 4.6	83.9 ± 19.0	77.8 ± 20.2	798 (37.41)	NA	NA	1,881 (88.19)	252 (11.81)	NA	NA	NA
	VATS	5975	66.65 ± 8.9	3,377 (56.52)	34.6 ± 4.5	83.1 ± 19.3	76.2 ± 20.0	2111 (35.33)			5,344 (89.44)	631 (10.56)			
Sezen 2023	RATS	27	62 ± 33	9 (33.33)	NA	NA	NA	NA	NA	NA	25 (92.59)	2 (7.41)	14 (51.85)	9 (33.33)	4 (14.81)
	VATS	95	60 ± 13	37 (38.95)							87 (91.58)	8 (8.42)	62 (65.26)	21 (22.11)	12 (12.63)
Pierluigi 2018	RATS	23	64.49 ± 30.90	NA	NA	88.21 ± 41.88	NA	NA	NA	50.03 ± 84.54	21 (91.3)	NA	NA	NA	NA
	VATS	42	62.04 ± 31.32			94.67 ± 67.55				32.99 ± 49.89	40 (97.6)				
Benjamin 2025	RATS	1995	66.5 ± 9.39	NA	NA	89.28 ± 20.48	74.16 ± 18.9	493 (24.71)	NA	NA	1,555 (77.94)	440 (22.06)	1,486 (75.16)	280 (14.16)	211 (10.67)
	VATS	3692	66.37 ± 9.4			88.69 ± 19.36	73.13 ± 18.13	723 (19.58)			2,923 (79.17)	769 (20.83)	2,714 (73.99)	589 (16.06)	365 (9.95)
Worrell 2018	RATS	25	68	13 (52.00)	NA	NA	NA	NA	NA	24.44 ± 28.30	25 (100)	NA	NA	NA	NA
	VATS	73	65	38 (52.05)						38.51 ± 65.80	73 (100)				
Fabbri 2023	RATS	403	70 ± 10	246 (61.04)	NA	90 ± 30	76 ± 26	74 (18.36)	42 (10.42)	29 ± 19	403 (100)	NA	313 (77.67)	77 (19.11)	13 (3.23)
	VATS	216	69 ± 10	139 (64.35)		92 ± 30	71 ± 25	54 (25.00)	31 (14.35)	28 ± 16	216 (100)		160 (74.07)	47 (21.76)	9 (4.17)
Duclos 2018	RATS	89	66.15 ± 10.55	39 (43.82)	25.06 ± 4.52	90.65 ± 20.35	65.24 ± 15.82	NA	NA	NA	NA	NA	61 (68.54)	11 (12.36)	2 (2.25)
	VATS	105	63.65 ± 9.77	48 (45.71)	25.41 ± 4.21	91.04 ± 18.04	67.17 ± 12.78						67 (63.81)	11 (10.48)	3 (2.86)
Cui 2020	RATS	4629	66.9 ± 9.9	2,624 (56.69)	NA	NA	NA	NA	NA	NA	4,629 (100)	0 (0)	2,736 (59.11)	993 (21.45)	900 (19.44)
	VATS	14279	66.6 ± 10.1	8,388 (58.74)							14,279 (100)	0 (0)	8,530 (59.74)	2,857 (20.01)	2,892 (20.25)
Dai 2018	RATS	45	59.8 ± 10.43	21 (46.67)	NA	NA	NA	NA	NA	27.6 ± 0.83	≥ 90	NA	NA	NA	NA
	VATS	45	59.4 ± 9.79	21 (46.67)						26.9 ± 0.76					
Chongwu 2019	RATS	36	57.2 ± 8.90	19 (52.78)	NA	89.75	NA	1 (2.78)	4 (11.11)	27.2 ± 6.80	36 (100) 85 (100)	0 (0)	33 (91.67)	2 (5.56)	1 (2.78)
	VATS	85	59.7 ± 8.80	47 (55.29)		95.81		3 (3.53)	9 (10.59)	27.3 ± 8.70		0 (0)	78 (91.76)	4 (4.71)	3 (3.53)
Tong 2019	RATS	49	61.80 ± 7.20	5 (10.20)	24.10 ± 3.10	NA	NA	NA	NA	NA	NA	NA	NA	41 (82.90)	8 (17.1)
	VATS	73	59.4 ± 8.8	9 (12.30)	23.80 ± 3.10									61 (83.30)	12 (16.7)
Kneuertz 2019	RATS	296	65.2 ± 11.2	161 (54.39)	27.9 ± 6.7	82.3 ± 20.3	76.1 ± 22.7	133 (44.93)	28 (9.46)	NA	NA	NA	252 (85)	NA	NA
	VATS	161	62.0 ± 10.4	86 (53.42)	27.8 ± 6.1	85.3 ± 19.0	86.9 ± 22.0	55 (34.16)	25 (15.53)				127 (79)		
Zhang 2024	RATS	148	55.00 ± 8.98	101 (68.24)	23.24 ± 2.4	96.86 ± 15.14	92.28 ± 15.97	NA	NA	NA	103 (69.59)	45 (30.41)	131 (88.51)	NA	NA
	VATS	148	56.74 ± 12.54	96 (64.86)	22.82 ± 2.5	98.10 ± 14.71	93.51 ± 17.01				86 (58.11)	62 (41.89)	133 (89.86)		
Wang 2024	RATS	102	56.95 ± 10.73	76 (74.51)	22.88 ± 2.54	2.43 ± 0.54	7.29 ± 1.57	NA	8 (7.84)	12.8 ± 6.5	0 (0)	102 (100)	102 (100.00)	NA	NA
	VATS	102	54.07 ± 12.52	71 (69.61)	22.99 ± 3.71	2.57 ± 0.63	7.68 ± 1.82		13 (12.75)	12.2 ± 5.3	0 (0)	102 (100)	102 (100.00)		
Shahoud 2024	RATS	78	65 ± 12	NA	28.7 ± 6.4	76.1 ± 30.2	70.6 ± 29.0	NA	NA	NA	78 (100)	0 (0)	NA	NA	NA
	VATS	50	70.3 ± 10		26.9 ± 6.2	78.1 ± 22.7	72.2 ± 33.1				50 (100)	0 (0)			
Barman 2025	RATS	42	64.64 ± 12.53	13 (30.95)	NA	86.68 ± 19.39	84.31 ± 48.75	NA	13 (30.95)	30.9 ± 23.3	NA	NA	16 (38.10)	6 (14.29)	20 (47.62)
	VATS	44	70.31 ± 8.89	27 (61.36)		69.82 ± 12.25	101.87 ± 21.98		16 (36.36)	36.9 ± 25.1			25 (56.81)	8 (18.18)	11 (25.00)
Zhan 2024	RATS	240	68.09 ± 9.14	139 (57.92)	25.77 ± 4.62	NA	NA	NA	NA	NA	240 (100)	0 (0)	178 (74.17)	34 (14.17)	28 (11.67)
	VATS	458	70.00 ± 8.92	279 (60.92)	25.93 ± 5.17						458 (100)	0 (0)	372 (81.22)	40 (8.73)	46 (10.04)
Zhou 2024	RATS	1190	56.5 ± 10.3	605 (50.84)	NA	NA	NA	NA	NA	21.6 ± 13.6	708 (59.50)	461 (38.74)	906 (76.13)	157 (13.19)	127 (10.67)
	VATS	868	58.4 ± 9.2	447 (51.50)						22.3 ± 11.7	569 (65.55)	299 (34.45)	676 (77.88)	106 (12.21)	86 (9.91)
Xingchi 2018	RATS	134	62.14 ± 8.6	67 (50.00)	NA	NA	NA	NA	NA	NA	134 (100)	0 (0)	104 (77.61)	11 (12.36)	2 (2.25)
	VATS	213	61.3 ± 8.0	95 (44.60)							213 (100)	0 (0)	137 (64.32)	11 (10.48)	3 (2.86)
Yang 2017	RATS	172	68.0 ± 10.2	98 (57)	26.8 ± 3.5	91.6 ± 17.4	84.9 ± 23.1	19 (11.0)	18 (10.5)	28 ± 11	172 (100)	0 (0)	139 (80.8)	33 (19.2)	0 (0)
	VATS	141	67.5 ± 10.0	88 (62)	27.1 ± 3.7	90.3 ± 17.9	85.4 ± 20.5	16 (11.3)	15 (10.6)	27 ± 10	141 (100)	0 (0)	115 (81.6)	26 (18.4)	0 (0)
Nelson 2019	RATS	106	67 ± 10	59 (56)	27.1 ± 3.8	86 ± 17	NA	18 (17.0)	19 (17.9)	30 ± 6.05	106 (100)	0 (0)	79 (74.5)	27 (25.5)	0 (0)
	VATS	301	67 ± 8	162 (54)	26.8 ± 3.5	86 ± 18		58 (19.3)	51 (17.0)	25 ± 4.03	301 (100)	0 (0)	247 (82.1)	54 (17.9)	0 (0)
Zhang 2020	RATS	257	53.5 ± 11.0	173 (67.3)	23.1 ± 2.7	NA	NA	2 (0.8)	NA	9.0 ± 4.0	0 (0)	257 (100)	256 (99.6)	NA	NA
	VATS	257	52.2 ± 11.9	168 (65.4)	23.0 ± 3.9			1 (0.4)		9.9 ± 5.5	0 (0)	257 (100)	255 (99.2%)	1 (0.4%)	

N, number; BMI, body mass index; FEV1, forced expiratory volume in 1 s; DLCO, diffusing capacity of the lung for carbon monoxide; COPD, chronic obstructive pulmonary disease; DM, diabetes mellitus; NA, not available.

**Table 2:** Results from 25 studies

Study ID (Year)	Group	Cases (*n*)	Number of dissected lymph node (mean ± SD)	Total morbidity *n* (%)	Drainage mL(Mean ± SD)	Atrial fibrillation *n* (%)	Pneumonia *n* (%)	Pneumothorax *n* (%)	Number of lymph node stations sampled (mean ± SD)	5-year survival rate *n* (%)	ICU stay days (mean ± SD)	30-day mortality *n* (%)	90-day mortality *n* (%)	Total cost (*thousand US$*)	R0 *n* (%)	R1 *n* (%)	Operative time minutes (Mean ± SD)	Blood loss mL (Mean ± SD)	Chest tube duration days (Mean ± SD)	Hospital stays days (Mean ± SD)	Conversion to Open *N* (%)
Giulia 2021	RATS	38	NA	22 (57.89)	NA	4 (10.53)	4 (10.53)	0 (0)	5.2 ± 1.4	NA	1.07 ± 0.12	NA	NA	NA	38 (100.00)	38 (100.00)	179 ± 54.2	NA	4.36 ± 2.31	5.31 ± 3.08	3 (7.89)
	VATS	39		13 (33.33)		3 (7.69)	1 (2.56)	1 (3)	3.9 ± 1.2		1.45 ± 0.33				38 (97.44)	38 (97.44)	183 ± 40.9		4.57 ± 2.66	4.36 ± 2.31	2 (5.13)
Qianjun 2020	RATS	50	5.04 ± 3.96	6 (12.00)	NA	2 (4.00)	0 (0.00)	1 (2)	NA	50 (100)	1.36 ± 1.12	NA	NA	NA	NA	NA	89.62 ± 57.61	67.70 ± 38.17	4.58 ± 3.71	4.71 ± 4.58	NA
	VATS	80	6.29 ± 4.14	11 (13.75)		2 (2.50)	1 (1.25)	2 (2.5)		79 (98.75)	2.76 ± 1.23						115.40 ± 43.69	134.22 ± 88.50	6.04 ± 4.41	7.55 ± 6.41	
Rajwanth 2020	RATS	338	NA	95 (28.13)	NA	NA	NA	NA	NA	NA	NA	10 (3)	NA	28.71 ± 6.58	NA	NA	NA	NA	NA	NA	NA
	VATS	1230		429 (34.96)								17 (1.38)		26.03 ± 5.96							
Seder 2022	RATS	2133	NA	668 (31.31)	NA	NA	49 (2.30)	NA	NA	NA	NA	NA	NA	NA	NA	NA	NA	NA	NA	4.3 ± 3.8	69 (3.23)
	VATS	5975		1,961 (32.82)			182 (3.05)													7.1 ± 4.5	888 (14.86)
Sezen 2023	RATS	27	14 ± 11	1 (3.70)	125 ± 95	1 (3.70)	0 (0.00)	0 (0)	NA	NA	NA	NA	NA	NA	NA	NA	110 ± 50	NA	NA	4 ± 2	NA
	VATS	95	12 ± 8	16 (16.84)	150 ± 125	3 (3.16)	1 (1.05)	1 (1.3)									100 ± 60			5 ± 3	
Pierluigi 2018	RATS	23	NA	8 (34.78)	NA	NA	NA	NA	4.7 ± 0.97	NA	1.12 ± 0.26	1 (4.35)	NA	7.61 ± 3.61	NA	NA	155 ± 37.1	NA	NA	4.1 ± 2.20	2 (8.70)
	VATS	42		21 (50.00)					2.9 ± 1.10		1.86 ± 0.89	1 (2.38)		5.89 ± 2.15			186 ± 41.3			6.6 ± 6.20	5 (11.90)
Benjamin 2025	RATS	1995	NA	685 (34.34)	NA	NA	92 (4.61)	NA	NA	NA	NA	NA	39 (1.95)	NA	1,889 (96.62)	1,933 (96.83)	157.38 ± 63.23	NA	NA	7.61 ± 5.76	NA
	VATS	3692		1,285 (34.90)			214 (5.80)						83 (2.25)		3,450 (95.44)	3,537 (95.71)	151.92 ± 53.5			8.04 ± 6.76	
Worrell 2018	RATS	25	17.44 ± 8.30	6 (24.00)	NA	2 (8.00)	1 (4.00)	1 (4)	NA	19 (76.00)	NA	NA	NA	13.24 ± 3.24	NA	NA	231	116 ± 75	4.07 ± 3.93	NA	2 (8.00)
	VATS	73	13.94 ± 7.23	20 (27.40)		9 (12.33)	2 (2.74)	1 (1.4)		54 (73.97)				11.03 ± 2.27			183	86 ± 56	6.34 ± 4.39		9 (12.33)
Fabbri 2023	RATS	403	NA	137 (34.00)	NA	NA	NA	NA	7 ± 2	284 (70.5)	NA	1 (0.25)	1 (0.25)	NA	NA	NA	NA	NA	3.12 ± 2.3	5.11 ± 3.27	NA
	VATS	216		83 (38.43)					5 ± 2	148 (68.5)		0 (0.00)	0 (0.00)						3.56 ± 2.46	5.67 ± 3.86	
Duclos 2018	RATS	89	NA	35 (39.33)	NA	NA	NA	NA	NA	NA	NA	NA	1 (1.12)	NA	72 (80.89)	87 (97.75)	160 ± 45	NA	NA	4.85 ± 1.01	5 (5.61)
	VATS	105		44 (41.90)									2 (1.90)		80 (76.19)	104 (99.05)	143 ± 50			4.35 ± 2.26	10 (9.52)
Cui 2020	RATS	4629	NA	NA	NA	NA	NA	NA	NA	NA	NA	60 (1.30)	110 (2.38)	NA	NA	NA	NA	NA	NA	NA	364 (7.86)
	VATS	14279										157 (1.10)	288 (2.02)								2161 (15.13)
Dai 2018	RATS	45	22.67 ± 9.67	NA	275 ± 145.42	NA	NA	NA	6.31 ± 1.43	NA	NA	NA	NA	NA	NA	NA	NA	50.30 ± 32.33	10.55 ± 5.31	NA	NA
	VATS	45	15.51 ± 5.41		347.6 ± 125.8				4.91 ± 1.04									208.60 ± 132.63	11.45 ± 4.22		
Chongwu 2019	RATS	36	NA	NA	NA	NA	3 (8.33)	2 (5.6)	NA	NA	NA	NA	NA	NA	34 (94.44)	34 (94.44)	96.8 ± 23.00	NA	4.11 ± 1.18	NA	NA
	VATS	85					6 (7.06)	6 (7.1)							81 (95.29)	81 (95.29)	100.1 ± 37.60		4.44 ± 2.25		
Tong 2019	RATS	49	NA	NA	NA	2 (4.08)	2 (4.08)	0 (0)	NA	NA	NA	NA	0 (0)	NA	NA	NA	200.0 ± 73.30	118.9 ± 117.2	NA	7.70 ± 4.40	0 (0.00)
	VATS	73				2 (2.70)	5 (6.84)	3 (4.6)					5 (6.84)				291.5 ± 87.20	182.5 ± 134.6		10.20 ± 6.50	4 (5.4)
Kneuertz 2019	RATS	296	NA	136 (45.95)	NA	24 (8.11)	10 (3.38)	12 (4.4)	NA	NA	4.4 ± 1.2	3 (1.01)	NA	17.22 ± 7.32	NA	NA	283.6 ± 71.9	NA	NA	3.8 ± 0.84	41 (13.85)
	VATS	161		62 (38.51)		24 (14.9)	6 (3.73)	2 (1.9)			6.0 ± 1.6	1 (0.62)		17.25 ± 8.35			313.2 ± 74.6			5.0 ± 0.66	21 (13.04)
Zhang 2024	RATS	148	10.65 ± 5.24	19 (12.84)	297.77 ± 149.73	NA	8 (5.41)	10 (7)	6.00 ± 1.50	NA	NA	NA	NA	10.5 ± 0.89	NA	NA	91.49 ± 29.95	53.51 ± 7.49	3.22 ± 1.56	7.35 ± 2.25	NA
	VATS	148	8.35 ± 5.24	25 (16.89)	244.92 ± 160.21		4 (2.70)	8 (5.6)	4.65 ± 2.25					6.82 ± 1.32			87.81 ± 35.94	67.57 ± 37.43	3.86 ± 1.75	7.18 ± 1.87	
Wang 2024	RATS	102	NA	17 (16.70)	185.44 ± 109.14	NA	NA	1 (1)	NA	NA	NA	NA	NA	8.51 ± 0.92	NA	NA	58.59 ± 12.20	98.77 ± 51.50	NA	NA	NA
	VATS	102		41 (39.90)	268.7 ± 149.99			3 (2.9)						4.52 ± 1.15			66.12 ± 21.56	128.87 ± 65.79			
Shahoud 2024	RATS	78	NA	NA	NA	1 (1.28)	NA	14 (17.9)	6.1 ± 1.8	NA	NA	NA	NA	NA	NA	NA	163 ± 84	NA	NA	2.6 ± 1.6	1 (1.28)
	VATS	50				4 (8.00)		9 (18)	3.9 ± 1.5								170 ± 69			4.0 ± 4.8	0 (0.00)
Barman 2025	RATS	42	4.21 ± 4.17	11 (26.19)	NA	4 (9.52)	NA	NA	NA	NA	0.69 ± 2.7	1 (2.4)	NA	NA	NA	NA	NA	72.64 ± 218.2	NA	3.88 ± 3.26	NA
	VATS	44	3.81 ± 2.63	8 (18.18)		7 (15.91)					1 ± 5.1	2 (4.2)						208.97 ± 240.58		6.22 ± 5.82	
Zhan 2024	RATS	240	8.09 ± 6.15	NA	NA	NA	NA	NA	4.00 ± 1.49	NA	NA	NA	NA	NA	NA	NA	NA	NA	NA	2.81 ± 1.49	3 (1.25)
	VATS	458	2.35 ± 2.23						1.70 ± 1.49											2.83 ± 1.49	18 (3.93)
Zhou 2024	RATS	1190	NA	NA	273.4 ± 186.6	NA	NA	NA	NA	NA	NA	NA	NA	9.72 ± 1.95	NA	NA	132.6 ± 42.9	75.6 ± 80.7	3.7 ± 2.7	11.3 ± 5.0	13 (1.09)
	VATS	868			258.6 ± 177.8									6.3 ± 2.5			137.6 ± 60.2	116.6 ± 248.2	4.1 ± 2.5	10.5 ± 3.7	46 (5.30)
Xingchi 2018	RATS	134	18 ± 9	NA	NA	NA	NA	NA	NA	116 (86.57)	NA	NA	NA	NA	NA	NA	NA	49 ± 39	NA	NA	NA
	VATS	213	11 ± 8							155 (72.77)								202 ± 239			
Yang 2017	RATS	172	**9**± **1.77**	51 (29.7)	NA	NA	NA	NA	5 ± 1.33	134 (77.6)	NA	0 (0)	0 (0)	NA	170 (99)	NA	NA	NA	NA	NA	16 (9.3)
	VATS	141	**7**± **1.52**	35 (24.8)					3 ± 1.16	104 (73.5)		0 (0)	1 (0.7)		141 (100)						8 (5.7)
Nelson 2019	RATS	106	NA	NA	NA	7 (6.6)	7 (6.6)	NA	**5**± **1.51**	NA	0.3 ± 0.8	NA	1 (0.94)	32.1 ± 4.5	100 (100)	0 (0)	NA	100 ± 37.82	NA	4 ± 0.50	8 (7.54)
	VATS	301				25 (8.3)	24 (8)		**5**±**1.42**		0.5 ± 1.2		6(2)	26.1 ± 3.8	99 (99)	1 (0.3)		150 ± 50.81		4 ± 0.76	33 (11)
Zhang 2020	RATS	257	NA	46 (17.9)	NA	NA	13 (5.1)	11 (4.3)	NA	NA	NA	0 (0)	NA	12.01 ± 1.67	257 (100)	0 (0)	147.9 ± 52.4	50 ± 12.7	NA	NA	1 (0.4)
	VATS	257		38 (14.8)			10 (3.9)	5 (1.9)				0 (0)		7.83 ± 1.29	256 (99.6)	1 (0.4)	149.2 ± 49.7	100 ± 17.8			3 (1.2)

Bolded terms represent key surgical and oncologic quality indicators. Conversion to OPEN denotes intraoperative conversion to thoracotomy. R0 indicates complete resection with negative margins, whereas R1 indicates microscopically positive margins.

RATS, robotic-assisted thoracic surgery; VATS, video-assisted thoracoscopic surgery; ICU, intensive care unit; ; SD, standard deviation; NA, not available.

Data presentation: Continuous variables are presented as mean ± SD; categorical variables as n (%). Total cost is reported in thousand US dollars (USD).

Outcome definitions: 30-day and 90-day mortality refer to postoperative mortality within 30 and 90 days, respectively; 5-year survival refers to 5-year overall survival.

**Figure 2:**
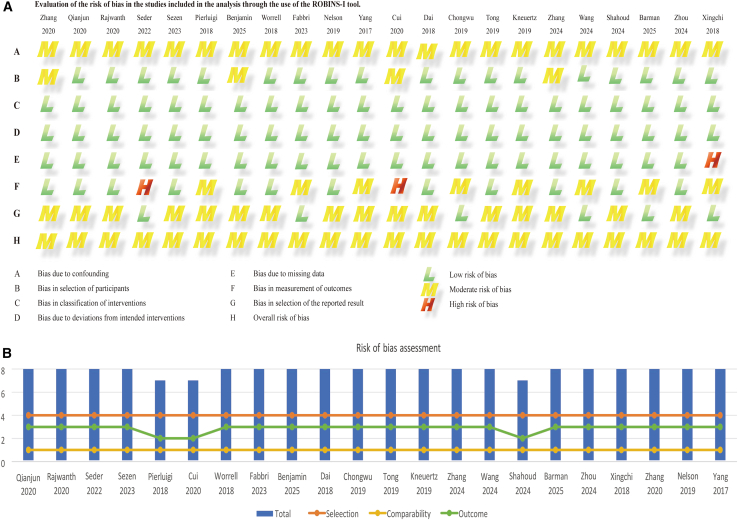
Risk of bias assessment of included studies (A and B) Risk of bias assessment. Evaluation of the risk of bias in the studies included in the analysis through the use of the ROBINS-I tool.

**Figure 3:**
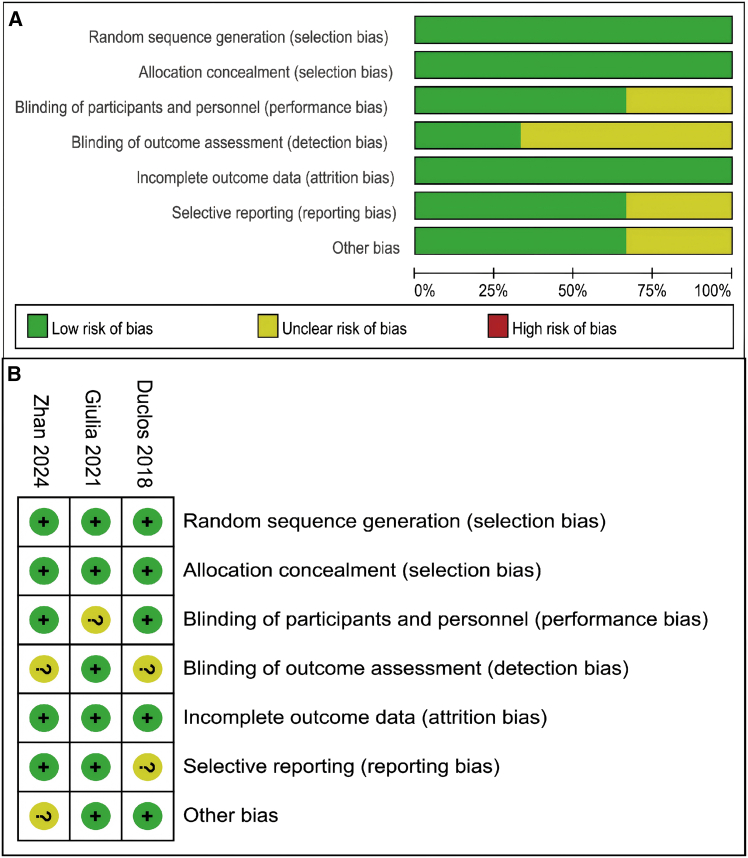
Quality assessment of randomized controlled trials (A and B) Quality of RCTs according to the cochrane collaboration manual. Red: high risk; yellow: unclear risk; green: low risk.

The risk of bias assessment is shown in Figure 2A. Overall, most studies had a low risk of bias, although some exhibited methodological limitations. A study-level evaluation is provided in Figure 2B, where the ROBINS-I tool indicated a moderate risk of bias. In addition, three RCTs were assessed using the Cochrane Handbook, and their methodological quality was rated as good (Figures 3A and 3B).

### Number of lymph node stations sampled

**Figure 4:**
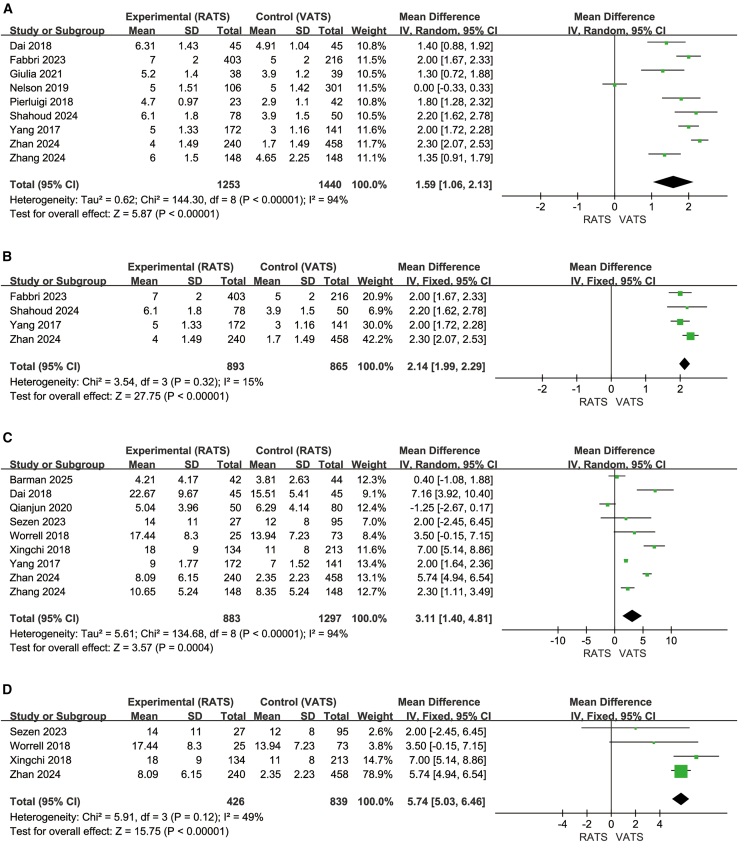
Lymph node sampling and dissection outcomes (A–D) (A and B) Number of lymph node stations sampled and lobectomy subgroup; (C and D) number of dissected lymph nodes and lobectomy subgroup.

Nine studies in the overall analysis (1,253 RATS, 1,440 VATS) (Figure 4A)^5^^,^^20^^,^^25^^,^^28^^,^^34^^,^^36^^,^^38^^,^^41^^,^^43^ and four studies in the lobectomy subgroup (893 RATS, 865 VATS) (Figure 4B)^28^^,^^34^^,^^35^^,^^36^ consistently showed that RATS yielded more sampled lymph node stations than VATS. In the overall analysis, RATS demonstrated a significant advantage (MD = 1.59, 95% CI, 1.06–2.13; Z = 5.87, *p* < 0.00001) despite high heterogeneity (I^2^ = 94%). In the lobectomy subgroup, this superiority remained stable (MD = 2.14, 95% CI, 1.99–2.29; Z = 27.75, *p* < 0.00001) with low heterogeneity (I^2^ = 15%). Across all studies, mean differences consistently ranged from approximately 1.30 to 2.30, favoring RATS.

### Number of dissected lymph nodes

Nine studies in the overall analysis (883 RATS, 1,297 VATS) (Figure 4C)^5^^,^^21^^,^^24^^,^^27^^,^^34^^,^^37^^,^^38^^,^^40^^,^^41^ and four studies in the lobectomy subgroup (426 RATS, 839 VATS) (Figure 4D)^24^^,^^27^^,^^34^^,^^40^ consistently showed that RATS dissected more lymph nodes than VATS. In the overall analysis, RATS demonstrated a significant advantage (MD = 3.11, 95% CI, 1.40–4.81; Z = 3.57, *p* = 0.0004) despite high heterogeneity (I^2^ = 94%). In the lobectomy subgroup, this superiority remained marked (MD = 5.74, 95% CI, 5.03–6.46; Z = 15.75, *p* < 0.00001) with moderate heterogeneity (I^2^ = 49%). Across all included studies, the mean differences consistently favored RATS.

### Blood loss

**Figure 5:**
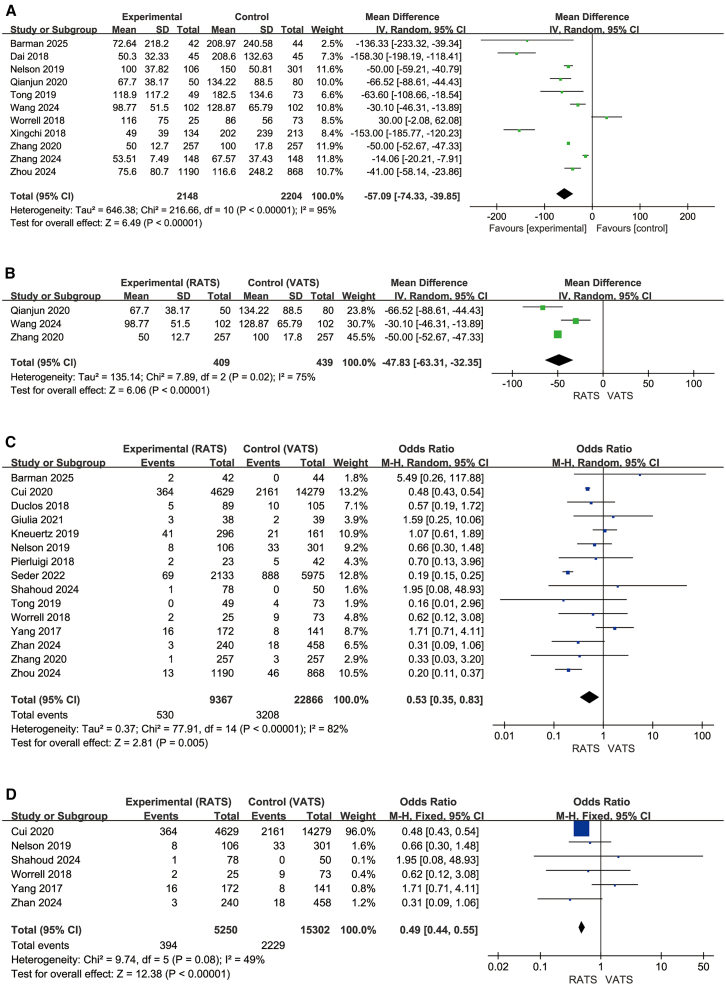
Intraoperative blood loss and surgical conversion (A–D) (A and B) Blood loss and segmentectomy subgroup; (C and D) conversion to open surgery and lobectomy subgroup.

Eleven studies in the overall analysis (2,148 RATS, 2,204 VATS) (Figure 5A)^21^^,^^27^^,^^32^^,^^35^^,^^37^^,^^38^^,^^39^^,^^40^^,^^41^^,^^42^^,^^43^ and three studies in the segmentectomy subgroup (409 RATS, 439 VATS) (Figure 5B)^21^^,^^35^^,^^42^ evaluated intraoperative blood loss. In the overall analysis, RATS showed significantly lower blood loss than VATS (MD = −57.09 mL, 95% CI, −74.33 to −39.85; Z = 6.49, *p* < 0.00001), with high heterogeneity (I^2^ = 95%). In the segmentectomy subgroup, RATS also demonstrated reduced blood loss (MD = −47.83 mL, 95% CI, −63.31 to −32.35; Z = 6.06, *p* < 0.00001), and heterogeneity was again high (I^2^ = 75%). Overall, most studies favored RATS, but the substantial heterogeneity in both analyses indicates that the magnitude of the pooled effect should be interpreted with caution.

### Conversion to open surgery

Fifteen studies in the overall analysis (9,367 RATS, 22,866 VATS) (Figure 5C)^5^^,^^20^^,^^23^^,^^25^^,^^27^^,^^29^^,^^30^^,^^32^^,^^33^^,^^34^^,^^36^^,^^37^^,^^39^^,^^42^^,^^43^ and six studies in the lobectomy subgroup (5,250 RATS, 15,302 VATS) (Figure 5D)^5^^,^^27^^,^^30^^,^^36^^,^^42^^,^^43^ evaluated conversion to thoracotomy. In the overall analysis, RATS showed a significantly lower conversion rate compared with VATS (OR = 0.53, 95% CI, 0.35–0.83; Z = 2.81, *p* = 0.005), with high heterogeneity (I^2^ = 82%). In the lobectomy subgroup, RATS also demonstrated a reduced conversion rate (OR = 0.49, 95% CI, 0.44–0.55; Z = 12.38, *p* < 0.00001), and heterogeneity was moderate (I^2^ = 49%). Across both analyses, most studies favored RATS.

### Operative time

**Figure 6:**
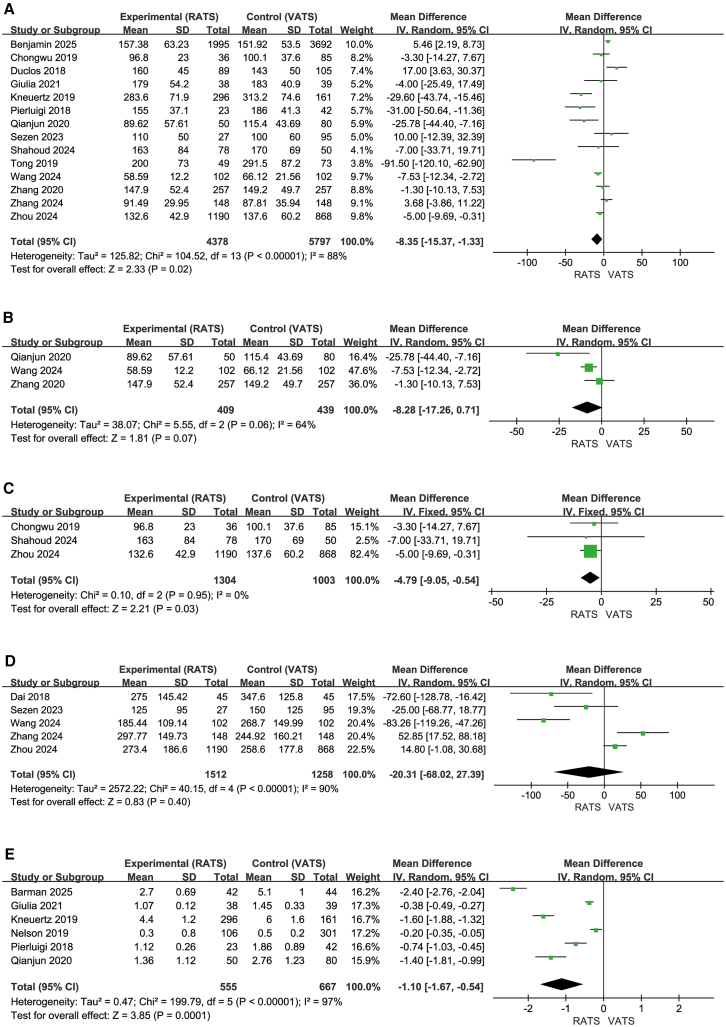
Operative characteristics and perioperative recovery (A–D) (A–C) Operative time, segmentectomy subgroup and lobectomy subgroup; (D) drainage volume; (E) ICU stay.

Fourteen studies in the overall analysis (4,378 RATS, 5,797 VATS) (Figure 6A)^20^^,^^21^^,^^24^^,^^25^^,^^26^^,^^29^^,^^31^^,^^32^^,^^33^^,^^35^^,^^36^^,^^38^^,^^39^^,^^42^ evaluated operative time. RATS showed a shorter operative time compared with VATS (MD = −8.35, 95% CI, −15.37 to −1.33; Z = 2.33, *p* = 0.02), with high heterogeneity (I^2^ = 88%). In the segmentectomy subgroup (409 RATS, 439 VATS) (Figure 6B),^21^^,^^35^^,^^42^ operative time tended to be shorter in the RATS group, but the difference was not statistically significant (MD = −8.28, 95% CI, −17.26 to 0.71; Z = 1.81, *p* = 0.07), and heterogeneity was moderate to high (I^2^ = 64%). In the lobectomy subgroup (1,304 RATS, 1,003 VATS) (Figure 6C),^31^^,^^36^^,^^39^ RATS demonstrated a significantly shorter operative time (MD = −4.79, 95% CI, −9.05 to −0.54; Z = 2.21, *p* = 0.03), with no observed heterogeneity (I^2^ = 0%).

### Drainage volume

Five studies (1,512 RATS, 1,258 VATS) (Figure 6D)^24^^,^^35^^,^^38^^,^^39^^,^^41^ evaluated postoperative drainage volume. There was no significant difference between RATS and VATS (MD = −20.31 mL, 95% CI, −68.02 to 27.39; Z = 0.83, *p* = 0.40). Heterogeneity was high (I^2^ = 90%).

### ICU stay

Six studies (555 RATS, 667 VATS) (Figure 6E)^20^^,^^21^^,^^25^^,^^33^^,^^37^^,^^43^ showed that ICU stay was shorter in the RATS group (MD = −1.10 days, 95% CI, −1.67 to −0.54; Z = 3.85, *p* = 0.0001). Heterogeneity was extremely high (Tau^2^ = 0.47; Chi^2^ = 199.79, df = 5, *p* < 0.00001; I^2^ = 97%), and therefore, a random-effects model was applied. Given the considerable variability across studies, the observed reduction in ICU stay should be interpreted with caution, although the overall trend favors RATS.

### R0–R1 resection outcomes

**Figure 7:**
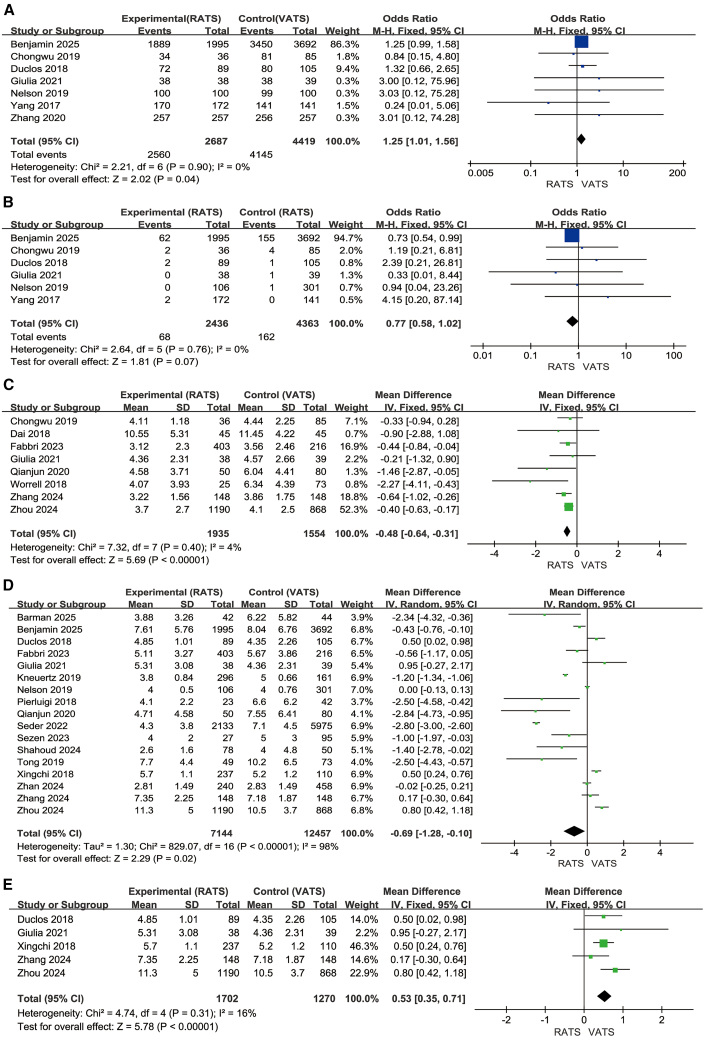
Surgical margins and postoperative recovery outcomes (A–E) (A) R0 resection; (B) R1 resection; (C) chest tube duration; (D and E) length of hospital stay and its subgroup analysis.

Thirteen studies evaluated margin status, including seven studies for R0 resection (2,687 RATS, 4,419 VATS) (Figure 7A)^5^^,^^20^^,^^26^^,^^29^^,^^31^^,^^42^^,^^43^ and six for R1 resection (2,436 RATS, 4,363 VATS) (Figure 7B)^5^^,^^20^^,^^26^^,^^29^^,^^31^^,^^43^^.^ RATS was associated with a significantly higher likelihood of achieving R0 resection compared with VATS (OR = 1.25, 95% CI, 1.01–1.56; Z = 2.02, *p* = 0.04), with no heterogeneity (I^2^ = 0%). For R1 resection, the pooled effect showed no statistically significant difference between the two approaches (OR = 0.77, 95% CI, 0.58–1.02; Z = 1.81, *p* = 0.07). Although the point estimate was numerically lower in the RATS group, the confidence interval crossed unity, indicating that current evidence does not confirm a reduction in R1 resection. A random-effects model using the Paule-Mandel estimator with Knapp-Hartung adjustment was applied to ensure robustness under minimal heterogeneity. Overall, RATS demonstrates a clear advantage in achieving R0 resection, whereas evidence regarding R1 reduction remains inconclusive.

### Chest tube duration

Eight studies (1,935 RATS, 1,554 VATS) (Figure 7C)^20^^,^^21^^,^^27^^,^^28^^,^^31^^,^^38^^,^^39^^,^^41^ evaluated chest tube duration. RATS was associated with a significantly shorter duration compared with VATS (MD = −0.48 days, 95% CI, −0.64 to −0.31; Z = 5.69, *p* < 0.00001). Heterogeneity was minimal (Chi^2^ = 7.32, df = 7, *p* = 0.40; I^2^ = 4%), supporting the use of a fixed-effect model and indicating highly consistent findings across studies.

### Length of hospital stay

Seventeen studies (7,144 RATS, 12,457 VATS) (Figure 7D)^20^^,^^21^^,^^23^^,^^24^^,^^25^^,^^26^^,^^28^^,^^29^^,^^32^^,^^33^^,^^34^^,^^36^^,^^37^^,^^38^^,^^39^^,^^40^^,^^43^ reported postoperative length of hospital stay. The pooled analysis indicated that RATS was associated with a slightly shorter hospital stay than VATS (MD = −0.69 days, 95% CI, −1.28 to −0.10; Z = 2.29, *p* = 0.02), although heterogeneity was substantial (I^2^ = 98%).

A predefined subgroup analysis was performed among studies in which surgeons explicitly followed the IASLC-recommended protocol for systematic lymph node dissection (sampling ≥6 stations, including ≥3 mediastinal stations) and restricted the population to stage I–II patients. Five studies (1,702 RATS, 1,270 VATS) (Figure 7E)^20^^,^^29^^,^^38^^,^^39^^,^^40^ fulfilled these surgical criteria. In this subgroup—representing procedures conducted under a more standardized lymphadenectomy framework—RATS showed a consistently shorter hospital stay (MD = 0.53 days, 95% CI, 0.35–0.71; Z = 5.78, *p* < 0.00001), with low heterogeneity (I^2^ = 16%). This suggests that when operations adhere to a uniform lymph node dissection standard, the difference in hospital stay becomes more homogeneous and consistently favors RATS.

### Postoperative complications

**Figure 8:**
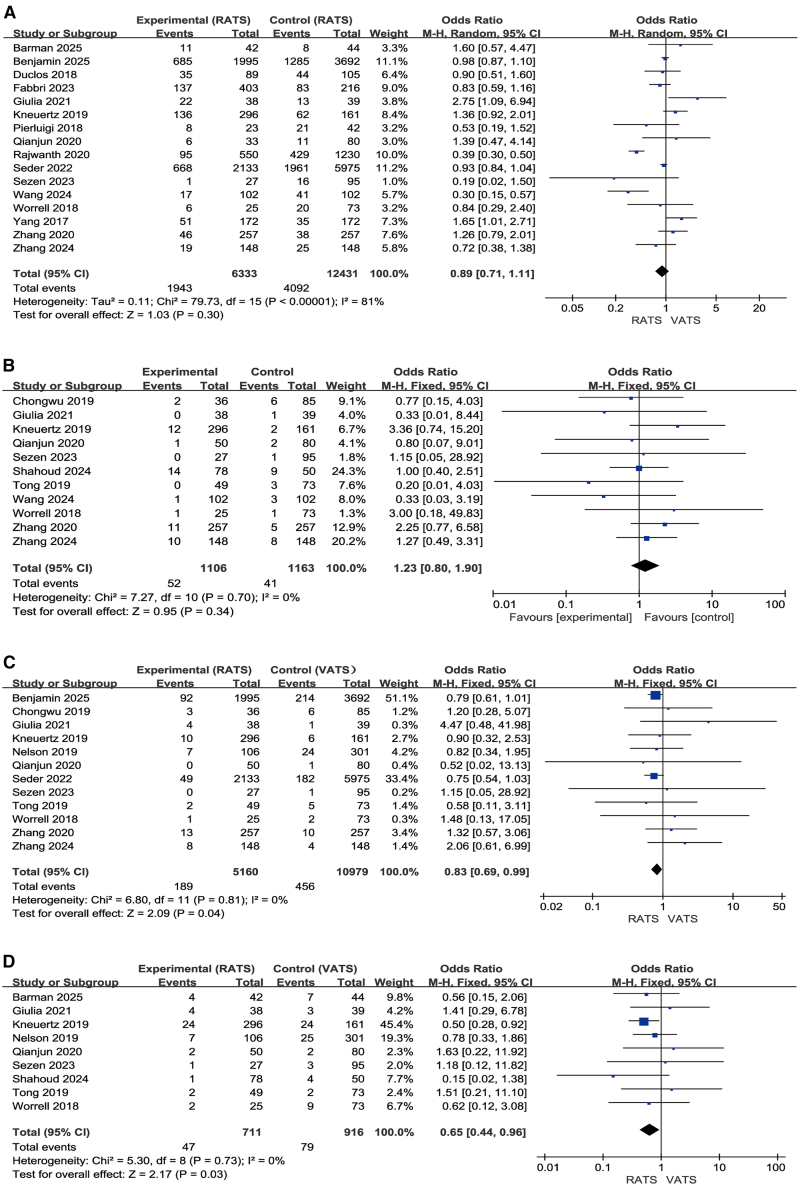
Postoperative complication outcomes (A–D) (A) Total postoperative complications; (B) pneumothorax; (C) pneumonia; (D) atrial fibrillation.

A total of 16 studies (6,333 RATS, 12,431 VATS) (Figure 8A),^5^^,^^20^^,^^21^^,^^22^^,^^23^^,^^24^^,^^25^^,^^26^^,^^27^^,^^28^^,^^29^^,^^33^^,^^35^^,^^37^^,^^38^^,^^42^ reported overall postoperative complications. The pooled effect showed no significant difference between RATS and VATS (OR = 0.89, 95% CI, 0.71–1.11; Z = 1.03, *p* = 0.30), with substantial heterogeneity was observed (I^2^ = 82%), a random-effects model was applied. Given the high heterogeneity observed in the overall complication analysis and the clinical relevance of certain postoperative events that are commonly encountered after thoracic surgery, additional subgroup analyses were conducted. These aimed to further elucidate specific perioperative risks and potential benefits associated with each surgical approach, focusing on three common complications: pneumothorax, pneumonia, and atrial fibrillation.

For pneumothorax, based on 11 studies (1,106 RATS, 1,163 VATS) (Figure 8B),^20^^,^^21^^,^^24^^,^^27^^,^^31^^,^^32^^,^^33^^,^^35^^,^^36^^,^^38^^,^^42^ there was no significant difference between groups (OR = 1.23, 95% CI, 0.80–1.90; *p* = 0.34; I^2^ = 0%). For pneumonia, based on 12 studies (5,160 RATS, 10,979 VATS) (Figure 8C),^20^^,^^21^^,^^23^^,^^24^^,^^26^^,^^27^^,^^31^^,^^32^^,^^33^^,^^38^^,^^42^^,^^43^ RATS showed a lower risk (OR = 0.83, 95% CI, 0.69–0.99; Z = 2.09, *p* = 0.04; I^2^ = 0%). For atrial fibrillation, based on nine studies (711 RATS, 916 VATS) (Figure 8D),^20^^,^^21^^,^^24^^,^^27^^,^^32^^,^^33^^,^^36^^,^^37^^,^^43^ RATS was also associated with a reduced incidence (OR = 0.65, 95% CI, 0.44–0.96; Z = 2.17, *p* = 0.03; I^2^ = 0%). Taken together, these findings suggest that while the incidence of postoperative atelectasis is comparable between RATS and VATS, RATS may confer advantages in reducing the risks of postoperative pneumonia and atrial fibrillation, indicating a potentially more favorable cardiopulmonary recovery profile and improved perioperative outcomes.

### Total hospitalization expenses

**Figure 9:**
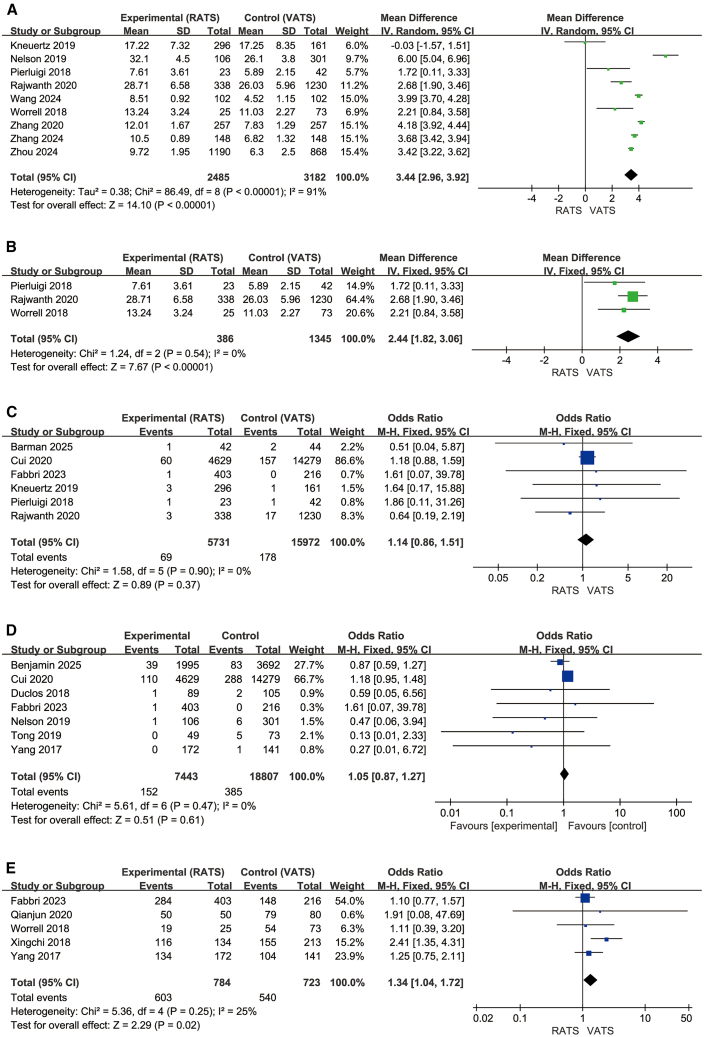
Hospitalization costs, mortality, and long-term survival (A–D) (A and B) Total hospitalization expenses and its subgroup analysis; (C and D) short-term mortality (30 days and 90 days); (E) 5-year overall survival.

Nine studies (2,485 RATS, 3,182 VATS) (Figure 9A)^22^^,^^25^^,^^27^^,^^33^^,^^35^^,^^38^^,^^39^^,^^42^^,^^43^ reported total cost. RATS was associated with significantly higher costs than VATS (MD = 3.44, 95% CI, 2.96–3.92; Z = 14.10, *p* < 0.00001), with substantial heterogeneity (I^2^ = 91%). A subgroup analysis was conducted for studies in which the median patient age exceeded 65 years (three studies; 386 RATS, 1,345 VATS) (Figure 9B).^22^^,^^25^^,^^27^ In this elderly subgroup, RATS remained more costly (MD = 2.44, 95% CI, 1.82–3.06; Z = 7.67, *p* < 0.00001), with no observed heterogeneity (I^2^ = 0%). Overall, both the general and elderly subgroups consistently showed higher total costs associated with RATS.

### Short-term mortality and long-term survival

Six studies (5,731 RATS, 15,972 VATS) (Figure 9C)^22^^,^^25^^,^^28^^,^^30^^,^^33^^,^^37^ reported 30-day mortality. No significant difference was observed between RATS and VATS (OR = 1.14, 95% CI, 0.86–1.51; Z = 0.89, *p* = 0.37; I^2^ = 0%). Similarly, seven studies (7,443 RATS, 18,807 VATS) (Figure 9D)^5^^,^^26^^,^^28^^,^^29^^,^^30^^,^^32^^,^^43^ evaluating 90-day mortality showed no difference between the two approaches (OR = 1.05, 95% CI, 0.87–1.27; Z = 0.51, *p* = 0.61; I^2^ = 0%).

Five studies were included, comprising 784 RATS (603 survival events) and 723 VATS (540 survival events) (Figure 9E),^5^^,^^21^^,^^27^^,^^28^^,^^40^ Heterogeneity was low (I^2^ = 25%), indicating good consistency among the included studies; therefore, a fixed-effects model was applied. The pooled results demonstrated that RATS was associated with a statistically significant improvement in 5-year overall survival compared with VATS, with an overall odds ratio of 1.34 (95% CI, 1.04–1.72, *p* = 0.02).

Taken together with the short-term mortality results—where 30- and 90-day mortality rates were comparable between the two approaches—these findings indicate that although RATS and VATS achieve similar short-term safety outcomes, RATS may confer a long-term survival benefit.

## Discussion

This meta-analysis included 25 studies involving 41,417 patients (12,645 RATS and 28,772 VATS) and evaluated 19 clinically relevant perioperative, oncologic, survival, and economic outcomes. Overall, the findings suggest that RATS is at least as safe as VATS and may provide specific advantages in technically demanding situations, particularly with respect to lymph node assessment, conversion to thoracotomy, recovery trajectories, and long-term survival, albeit at higher cost and with important contextual caveats.

### Technical performance and perioperative safety

Across the pooled analyses, RATS was associated with a lower conversion-to-thoracotomy rate and reduced intraoperative blood loss, while maintaining short-term mortality comparable to VATS. These patterns are consistent with the intrinsic features of robotic platforms—three-dimensional magnified vision, tremor filtration, and wristed instruments—which facilitate precise dissection around hilar and mediastinal vessels. In clinical practice, this may translate into a greater ability to complete minimally invasive resections in patients with dense adhesions, prior thoracic surgery, or complex hilar anatomy, circumstances where VATS is more likely to fail. Importantly, subgroup analyses based on anatomical resection type (lobectomy and segmentectomy) demonstrated consistent reductions in intraoperative blood loss and conversion-to-thoracotomy rates, indicating that these technical advantages are preserved across different anatomical extents of resection.

Operative time showed a more heterogeneous picture. Although the pooled estimate favored RATS in the current analysis, between-study variability was substantial, and individual trials reported divergent results. This inconsistency is plausibly explained by differences in the stage of robotic program maturation, team experience, and platform generation. Early in the learning curve, docking and troubleshooting can offset the technical efficiency of RATS, whereas high-volume centers often report operative times approaching or surpassing VATS. Because most included studies did not explicitly quantify learning-curve phases, the observed advantage in operative time should be interpreted cautiously and cannot be assumed to generalize to all institutions. Future prospective work incorporating objective learning-curve metrics (e.g., CUSUM-based thresholds) is needed to clarify how institutional experience modifies the time-efficiency of RATS. Similarly, operative-time subgroup analyses restricted to lobectomy and segmentectomy showed consistent directional trends, supporting that the heterogeneity in operative time is unlikely driven by resection type alone.

### Lymphadenectomy and oncologic radicality

One of the clearest signals in this meta-analysis is the superiority of RATS in lymph node assessment. RATS retrieved more nodal stations and a greater total number of nodes, and was associated with a modest increase in R0 resection rates. These findings support the concept that robotic systems facilitate more thorough mediastinal lymphadenectomy and more precise hilar dissection, which can improve pathological staging and the likelihood of complete tumor clearance. In subgroup analyses focused on lobectomy and segmentectomy, the advantages of RATS in lymph node station retrieval and total node counts remained consistent, indicating that the improved lymphadenectomy performance is stable across different anatomical resection scopes.

However, the apparent inconsistency between improved R0 rates and the borderline signal for R1 resections warrants a nuanced interpretation. R1 events were rare, and margin definitions, specimen handling, and pathological assessment protocols varied widely across studies. Under such conditions, pooled R1 estimates are highly sensitive to small changes and methodological differences. As a result, the current evidence is insufficient to claim a definitive advantage or disadvantage of RATS with respect to microscopic margin positivity. The more robust message is that RATS appears to facilitate systematic nodal dissection and slightly increases the probability of achieving R0 resection, but does not yet conclusively establish superior oncologic radicality. Future studies using standardized pathological protocols, central review, or sensitivity analyses that exclude studies with unclear margin definitions will be essential to refine this conclusion.

### Recovery trajectory and postoperative complications

With regard to postoperative recovery, RATS was associated with shorter chest tube duration, reduced ICU stay, and shorter overall hospital stay. These advantages likely reflect a combination of factors, including improved visualization of the pleural space, more controlled dissection with less chest wall and mediastinal trauma, and better hemostasis.

Notably, the only subgroup analysis available for recovery outcomes was the IASLC-compliant subgroup for length of stay, in which the benefit of RATS remained consistent and heterogeneity was markedly reduced, suggesting that standardized lymphadenectomy may enhance perioperative recovery patterns.

Importantly, while overall complication rates were similar between RATS and VATS, RATS was linked to lower incidences of postoperative pneumonia and atrial fibrillation. Both complications are tightly linked to perioperative respiratory mechanics, mediastinal manipulation, and systemic inflammatory response; thus, the reduced rates observed with RATS are biologically plausible and clinically meaningful. In contrast, there was no significant difference in postoperative pneumothorax, and the confidence intervals comfortably encompassed the null. These data support the interpretation that RATS does not increase respiratory complications overall and that the risk of pneumothorax, specifically, should be considered comparable between the two approaches. Standardized definitions and grading systems for complications in future studies will be important to further disentangle these patterns, particularly in high-risk subgroups.

### Short-term mortality and long-term survival

Short-term safety appears equivalent between RATS and VATS: both 30- and 90-day mortality rates were low and did not differ significantly. This is reassuring, given that short-term mortality is a composite reflection of patient selection, institutional processes, and surgical technique.

More notably, the analysis of 5-year overall survival suggested a potential long-term benefit associated with RATS. The pooled odds ratio favored RATS, with low-to-moderate heterogeneity, and the direction of effect aligns with the observed improvements in lymph node dissection quality and R0 resection rates. Nevertheless, the number of studies reporting long-term outcomes remains limited, and follow-up durations and adjuvant treatment strategies were not uniform. Consequently, although the survival signal is encouraging, it should be regarded as hypothesis-generating rather than definitive. Large, prospective, stage-stratified studies with standardized adjuvant therapy and long-term follow-up are necessary to confirm whether the technical advantages of RATS translate into durable survival gains.

### Heterogeneity, learning curve, and methodological considerations

Several continuous outcomes—including blood loss, ICU stay, and hospital stay—exhibited high I^2^ values, indicating substantial between-study heterogeneity. This is unsurprising in a pooled analysis that spans multiple countries, time periods, and institutional pathways. Differences in case mix (central vs. peripheral tumors, disease stage, comorbidities), perioperative management (including ERAS implementation), and definitions or timing of outcomes all likely contributed. Although random-effects models with REML and Knapp-Hartung adjustments were applied, and sensitivity analyses generally supported the robustness of the direction of effect, the magnitude of benefit should be interpreted with caution. Prediction intervals that span both benefit and no effect further underscore that RATS advantages may not be uniformly reproducible in all practice environments.

The learning curve is another under-appreciated modifier. Many included cohorts likely captured early and intermediate phases of robotic adoption, whereas VATS programs were more mature. Without explicit adjustment for surgeon or institutional experience, residual confounding by expertise remains a concern and may bias results either in favor of or against RATS, depending on local context.

### Economic implications and patient selection

Economically, RATS was consistently associated with higher total hospitalization costs. This was expected, given the capital investment, maintenance fees, and instrument costs associated with robotic platforms. In the subgroup of studies with median patient age >65 years, the higher total cost associated with RATS persisted, suggesting that the economic profile observed in the main analysis also applies to older, potentially higher-risk populations. However, the degree to which this cost difference is acceptable or sustainable depends heavily on health-system structure, reimbursement models, case volume, and the proportion of complex cases. In high-volume centers where robotic systems are fully amortized and complex resections are common, the incremental cost of RATS may be offset by reductions in conversion rates, complications, or length of stay. In contrast, in lower-resource settings or for routine low-complexity peripheral lobectomies, VATS remains the more economical and accessible option.

Taken together, the present findings support a nuanced, indication-driven approach: RATS may be preferentially considered for anatomically complex, centrally located, or high-risk tumors where its technical advantages and potential oncologic benefits are most likely to yield meaningful clinical returns, while VATS remains highly appropriate for standard, low-complexity resections.

### Strengths

This study has several notable strengths. First, it represents one of the most comprehensive and up-to-date meta-analyses comparing RATS and VATS, encompassing 25 studies and 41,417 patients, and offering extensive evaluation across 19 perioperative, oncologic, survival, and economic endpoints. Second, the analytical framework incorporated rigorous, pre-specified subgroup analyses, focusing on anatomical resection types, guideline-concordant lymphadenectomy, and age-related economic outcomes, thereby improving the granularity and interpretability of the results. Third, the inclusion of high-impact oncologic metrics—such as R0 and R1 resection rates, lymph node station retrieval, and long-term survival—provides meaningful insights into oncologic quality, an area where previous reviews have been limited. Fourth, the use of advanced random-effects estimators, including REML and Paule-Mandel, with Knapp-Hartung adjustments, enhances the robustness of variance estimation across heterogeneous studies. Finally, transforming key effect sizes into clinically interpretable measures (e.g., 53% reduction in conversion, 25% increase in R0 resection, 34% improvement in 5-year survival) strengthens the translational relevance and aids clinical decision-making.

### Limitations

Several limitations should be acknowledged. First, the evidence base was predominantly observational; although many studies used propensity score matching or weighting, residual confounding and unmeasured covariates cannot be excluded. Second, substantial heterogeneity remained for several continuous outcomes due to differences in case mix, surgical expertise, perioperative pathways, and reporting standards. Despite the use of advanced random-effects models, this heterogeneity reduces the precision of pooled estimates. Third, definitions of postoperative complications and pathological margins were inconsistent across studies, and the rarity of R1 events limited the reliability of margin-related outcomes. Fourth, long-term survival and economic outcomes were reported in only a subset of studies, preventing deeper stratification (e.g., tumor biology and adjuvant therapy) and reducing certainty in these estimates. Fifth, the procedural composition of included cohorts was overwhelmingly dominated by lobectomy (>90%), whereas segmentectomy accounted for ∼8.9% and wedge resections were rare and often intraoperative adaptations. Consequently, the pooled results primarily reflect lobectomy populations, and their generalizability to less extensive parenchyma-sparing procedures is limited. Sixth, learning-curve effects—an important determinant of robotic performance—could not be systematically evaluated owing to scarce reporting. Finally, the conversion of medians to means and standard deviations, when required, may introduce estimation error, and publication bias could not be reliably assessed due to the limited number of studies for several outcomes.

Collectively, these limitations warrant cautious interpretation and highlight areas where future, higher-quality studies are needed.

## Resource availability

### Lead contact

Further information and requests for resources should be directed to and will be fulfilled by the lead contact, Junfeng Li (liwlny@163.com).

### Materials availability

This study did not generate new unique reagents.

### Data and code availability

• All data supporting the findings of this study were derived from previously published articles. The study-level extracted data used for the meta-analyses are provided in the Supplementary Materials. No new datasets were generated or deposited in public repositories.
• Statistical analyses were performed using Review Manager software (RevMan, version 5.4; Cochrane Collaboration), as listed in the key resources table. No custom code was generated in this study.
• This study did not generate any other new resources.

## Acknowledgments

The authors extend their sincere gratitude to Professor Yinbin Zheng and Professor Guojun Wu from the Beijing Anzhen Nanchong Hospital of Capital Medical University & Nanchong Central Hospital for their valuable assistance with statistical software application and manuscript preparation.

## Author contributions

G.S., R.W., and X.Z. contributed equally and share first authorship. They were responsible for study conception, design, data analysis, and manuscript drafting. J.X., J.C., and R.C. contributed to data collection, statistical analysis, and manuscript preparation. Y.W., G.S., and R.W. participated in literature review, data interpretation, and critical revisions of the manuscript. K.W., X.Z., and Y.D. provided methodological guidance, supervised the overall study process, and contributed to data verification. J.L. and G.S. provided senior supervision, critical review, and final approval of the manuscript. All authors read and approved the final version of the manuscript.

## Declaration of interests

The authors declare no competing interests.

## STAR★Methods

### Key resources table

**Table:** 

REAGENT or RESOURCE	SOURCE	IDENTIFIER
**Deposited data**		

MEDLINE (via PubMed)	National Library of Medicine	https://pubmed.ncbi.nlm.nih.gov
Embase	Elsevier	https://www.embase.com
Cochrane CENTRAL	Cochrane Library	https://www.cochranelibrary.com
PROSPERO	University of York	CRD420251118017
Web of Science Core Collection	Clarivate Analytics	https://www.webofscience.com
Scopus	Elsevier	https://www.scopus.com

**Software and algorithms**		

EndNote X9	Clarivate Analytics	https://endnote.com
Microsoft Excel (Office 365)	Microsoft	https://www.microsoft.com
PRISMA 2020 statement	PRISMA Group	http://www.prisma-statement.org
Review Manager (RevMan)	Cochrane Collaboration	Version 5.4, https://revman.cochrane.org
Adobe Photoshop	Adobe Inc.	Version 2023, https://www.adobe.com

**Other**		

ROBINS-I tool	Cochrane Bias Methods Group	https://www.riskofbias.info/welcome/robins-i-tool
Newcastle–Ottawa Scale (NOS)	Ottawa Hospital Research Institute	http://www.ohri.ca/programs/clinical_epidemiology/oxford.asp

### Experimental model and study participant details

This study is a systematic review and meta-analysis and does not involve experimental models, animals, or human subjects.

### Method details

#### Study design and registration

We conducted this systematic review and meta-analysis in accordance with the PRISMA 2020 statement, the Cochrane Handbook, and the Meta-analysis of Observational Studies in Epidemiology (MOOSE) reporting guidance. The protocol was prospectively registered in PROSPERO (CRD420251118017). Eligibility criteria, outcomes, and analytic decision rules (including model selection and sensitivity analyses) were defined *a priori* and followed throughout the study.

#### Eligibility criteria

Studies were considered eligible if they met the following criteria: (1) study design: randomized controlled trials or observational cohort studies; (2) population: patients undergoing surgical resection for pulmonary nodules or tumors, predominantly non-small cell lung cancer; (3) intervention: robotic-assisted thoracic surgery (RATS); (4) comparison: video-assisted thoracoscopic surgery (VATS); and (5) outcomes: reporting at least one predefined perioperative, postoperative, oncologic, or cost-related outcome that allowed extraction or calculation of effect estimates.

Studies were excluded if they were duplicate publications, non-comparative in design (including case reports, reviews, conference abstracts, meta-analyses, or single-arm studies), non-English language articles, animal or *in vitro* studies, or if insufficient data were available for quantitative synthesis. When multiple publications reported overlapping patient cohorts, only the most complete or most recent study was included to avoid duplicate data.

#### Information sources and search strategy

We conducted a systematic review and meta-analysis in accordance with PRISMA 2020. Randomized controlled trials and cohort studies were identified by searching PubMed, Embase, Web of Science, Scopus, and the Cochrane Library from inception through June 2025. Backward and forward citation searching was performed on July 2, 2025 using a reference-checking tool. We restricted the search to studies published in English. The search strategy combined free-text keywords and Medical Subject Headings (MeSH), including “thoracoscopic,” “robot-assisted,” and “non-small cell lung cancer (NSCLC).” The full search strategy is provided in Table S1.

### Quantification and statistical analysis

Both a qualitative and quantitative synthesis of the data was performed. The minimum number of studies to perform a meta-analysis was three.

All statistical analyses were performed using Review Manager software (RevMan, version 5.4; Cochrane Collaboration). For binary outcomes, pooled effect estimates were expressed as odds ratios (ORs) with 95% confidence intervals (CIs), whereas for continuous outcomes, pooled estimates were expressed as mean differences (MDs) with 95% CIs. When studies reported medians and interquartile ranges, means and standard deviations were estimated using established methods described by Luo, McGrath, and Shi.^15^^,^^16^^,^^17^ Sensitivity analyses were conducted to assess the impact of these transformations on pooled estimates and heterogeneity.

Statistical heterogeneity was evaluated using the I^2^ statistic, with values <25% indicating low heterogeneity, 25–50% moderate heterogeneity, and >50% high heterogeneity. Fixed-effects models were applied when heterogeneity was low to moderate (I^2^ ≤50%), whereas random-effects models were used when heterogeneity exceeded 50%. In cases of extreme heterogeneity (I^2^ >75%), prediction intervals were calculated, and additional sensitivity analyses were performed by excluding influential studies or studies with a higher risk of bias. All decision rules for model selection and sensitivity analyses were prespecified in the PROSPERO protocol, and no deviations from the registered analytic plan occurred.

The exact value of *n* for each analysis represents the number of included studies contributing data to that specific outcome and is reported in the corresponding forest plots and figure legends. Measures of central tendency and dispersion for continuous variables were summarized as means and standard deviations, or medians and interquartile ranges where originally reported, with corresponding confidence intervals provided for pooled estimates. All statistical tests were two-sided, and a *P* value <0.05 was considered statistically significant. Detailed statistical results, including pooled effect estimates, confidence intervals, heterogeneity statistics, and sensitivity analyses, are presented in the Results section, figures, and figure legends.

## Supplemental information

Table S1. PubMed detailed search strategies and results

Data S1. Original dataset and forest plot: RevMan 5.4 (.rm5) dataset used to generate the meta-analysis and forest plots
